# Moving in Semantic Space in Prodromal and Very Early Alzheimer's Disease: An Item-Level Characterization of the Semantic Fluency Task

**DOI:** 10.3389/fpsyg.2022.777656

**Published:** 2022-02-21

**Authors:** Aino M. Saranpää, Sasa L. Kivisaari, Riitta Salmelin, Sabine Krumm

**Affiliations:** ^1^Psychology and Logopedics, Faculty of Medicine, University of Helsinki, Helsinki, Finland; ^2^Department of Neuroscience and Biomedical Engineering, Aalto University, Aalto, Finland; ^3^University Department of Geriatric Medicine FELIX PLATTER, Basel, Switzerland; ^4^Faculty of Medicine, University of Basel, Basel, Switzerland

**Keywords:** semantic fluency, Alzheimer's disease, Mild Cognitive Impairment, t-SNE, verbal fluency, semantic memory

## Abstract

The semantic fluency task is a widely used clinical tool in the diagnostic process of Alzheimer's disease. The task requires efficient mapping of the semantic space to produce as many items as possible within a semantic category. We examined whether healthy volunteers (*n* = 42) and patients with early Alzheimer's disease (24 diagnosed with amnestic Mild Cognitive Impairment and 18 with early Alzheimer's dementia) take advantage of and travel in the semantic space differently. With focus on the animal fluency task, we sought to emulate the detailed structure of the multidimensional semantic space by utilizing word2vec-method from the natural language processing domain. To render the resulting multidimensional semantic space visually comprehensible, we applied a dimensionality reduction algorithm (t-SNE), which enabled a straightforward division of the semantic space into sub-categories. Moving in semantic space was quantified with the number of items created, sub-categories visited, and switches and returns to these sub-categories. Multinomial logistic regression models were used to predict the diagnostic group with these independent variables. We found that returning to a sub-category provided additional information, besides the number of words produced in the task, to differentiate patients with Alzheimer's dementia from both amnestic Mild Cognitive Impairment patients and healthy controls. The results suggest that the frequency of returning to a sub-category may serve as an additional aid for clinicians in diagnosing early Alzheimer's disease. Moreover, our results imply that the combination of word2vec and subsequent t-SNE-visualization may offer a valuable tool for examining the semantic space and its sub-categories.

## 1. Introduction

The semantic fluency task is widely used in clinical settings to identify difficulties in speech production, executive functioning, and semantic memory performance (Lezak et al., 2004). In this task, participants are asked to produce as many words as possible in a given semantic category (e.g., animals) and time frame. An interpretation of the task is that while naming items, individuals move in a mental semantic word space, where they jump from one item to another. Further, there is evidence that brain regions responsible for spatial navigation also represent moving on a conceptual word-level speaking for the existence of cognitive maps (Viganò and Piazza, 2020). The task is used for predicting early Alzheimer's disease (Henry et al., 2004), since it measures semantic memory processes, which are shown to be one of the first areas of cognition to exhibit decline in test performance prior to dementia (Amieva et al., 2008; Mistridis et al., 2015). To score task performance, number of correct words are calculated. This process of word production is widely studied in Alzheimer's disease, as patients diagnosed with it name fewer words compared to healthy controls (Troyer et al., 1998; Fagundo et al., 2008; Raoux et al., 2008; Price et al., 2012).

In the neurocognitive framework, research on the semantic fluency task has mostly focused on comparing general higher level categories, such as living vs. non-living (Kivisaari et al., 2012; Tyler et al., 2013; Krumm et al., 2021) and animals, fruits, tools, and vehicles (Clarke and Tyler, 2014; Kivisaari et al., 2019). Also finer-grained semantic performance within single semantic categories has been examined, with the categories divided further into sub-categories based on the researcher's own evaluation. For instance, Troyer et al. (1997) created their own method of manual evaluation of sub-categories to investigate two strategies of semantic fluency performance: (1) producing items inside a sub-category (e.g., pets) and (2) sub-category switching, that is, moving between sub-categories (e.g., from pets to farm animals). Both of these strategies are needed to efficiently produce correct items in the semantic fluency task. Regarding patients with Alzheimer's disease, studies have found that they not only name fewer words compared to healthy controls (Troyer et al., 1998; Fagundo et al., 2008; Raoux et al., 2008; Price et al., 2012), but they also create smaller (Troyer et al., 1998; Fagundo et al., 2008) and fewer sub-categories (Pekkala, 2004) and switch less between sub-categories (Fagundo et al., 2008; Raoux et al., 2008). Other studies have not found differences between sub-category sizes (Epker et al., 1999; Pekkala, 2004; Raoux et al., 2008) and switching behavior (Price et al., 2012) between prodromal or early Alzheimer's disease patients and cognitively normal controls. These inconsistencies have been explained by differences in the state of disease progression, demographic variables, sampling, and study design (March and Pattison, 2006; Raoux et al., 2008). Notably, even though the Troyer et al. (1997) method entails clear instructions for scoring the sub-categories and switching, some studies have questioned the method's validity (Epker et al., 1999). Further, the method does not provide an unambiguous cluster structure, since according to the scoring rules, words can belong to multiple clusters. This may be reasonable semantically (as many words can belong to two or more semantic categories), but could make it difficult to inspect the switching phenomenon in detail. Other manually created measures have also been presented, but they too suffer from issues such as low inter-rater reliability or insufficient test-retest reliability (Abwender et al., 2001). Furthermore, to our knowledge no studies have yet investigated whether switching and producing sub-categories have *independent* effects over and beyond the number of words, while simultaneously taking into account the highly correlated nature of these measures.

Semantic processing can also be investigated in the context of semantic features and their relatedness. These features can be modal such as visual or auditory item characteristics (e.g., “has a nose”), functional properties (e.g., “swims”) or encyclopedic information (e.g., “is a predator”; Ellis and Young, 2013). Same features often apply for different items, but an item can sometimes be recognized by one specific property, i.e., humans can conjure an image of a dog in their mind just by hearing its bark. In the human brain, the basis of semantic processing is thought to lie in the co-activation of specific distributed sensorimotor regions primarily responsible for processing and perceiving the relevant features in order to produce concepts such as a dog (Tyler et al., 2000; Tyler and Moss, 2001; Vigliocco et al., 2004; Patterson et al., 2007). But how do individuals differentiate between very similar concepts such as different breeds of dogs? One explanation for this dilemma is presented by Taylor et al. (2011) in the form of the Conceptual Structure Account, which assumes that semantic processing is structured according to the statistical properties of the item's features, and processing of semantic concepts corresponds to the co-activation of the concept's features. The statistical properties, feature correlation, and feature distinctiveness, differentiate between shared features (high in feature correlation, such as “has eyes”) and unique features (high in feature distinctiveness, such as “has a trunk”) with which humans are able to distinguish between items. The task of conceptual, i.e., semantic processing can therefore be described as the interaction of feature distinctiveness and correlation, which defines the information required to perform the task in question. A similar framework of semantic processing has been used in the natural language processing research domain, which aims to emulate human processing of language by applying statistical modeling of word co-occurrence with the help of machine learning and large text corpora (Nadkarni et al., 2011). One of these methods is the word2vec algorithm developed by Mikolov et al. (2013), which builds (typically) 300-dimensional vector representations of words based on the contexts in which they appear.

In the present study, we combine these two frameworks of semantic processing to shed more light on how the prodromal and early Alzheimer's disease patients move in the semantic space during the fluency task compared to control participants. To emulate the process of item processing as a co-activation of features, we use an internet-derived text corpus and the word2vec algorithm to extract feature-based vectors with rich semantic information (Mikolov et al., 2013). Thus, we achieve a representation of the semantic space, which consists of all words that the participants produced in the semantic animal fluency task. To visualize the semantic space and the possibly emerging sub-categories in a readily tangible and comprehensible manner, we use a data-driven dimensionality reduction method, the t-Distributed Stochastic Neighbor Embedding (t-SNE) (van der Maaten and Hinton, 2008), which does not require a priori knowledge on the structure of the semantic space. With t-SNE, we condense the 300-dimensional semantic space into a two-dimensional map, which allows for a straightforward visual inspection and labeling of semantic sub-categories. Alternatively, additional clustering algorithms, such as k-means, could be considered atop the t-SNE visualization (Taskesen and Reinders, 2016; Devassy et al., 2020; DeLise, 2021). However, some concerns have been presented on utilizing another clustering algorithm with t-SNE (van der Maaten and Hinton, 2008). An additional analysis using the k-means clustering can be found in the Supplementary Material. Finally, when condensing multidimensional feature-based information into a singular point which reflects the closeness of the item to all other items, we achieve a simple, unambiguous solution, where an item can be assigned to a single sub-category.

With the acquired, unambiguous sub-categories, we can quantify the efficiency of semantic processing and how the participants move in the semantic space. In addition to the number of words produced in the task, we acquire measures of semantic processing efficiency, such as the number of sub-categories named, switching, and returning to the sub-categories. We then evaluate whether patients diagnosed with very early Alzheimer's dementia (AD) or amnestic Mild Cognitive Impairment (aMCI) exhibit different strategies in how they move in the semantic space compared to control participants. Finally, we examine whether using the sub-category and switching dimensions in addition to the number of the words the participants produce brings valuable information into the diagnostic process of prodromal and very early Alzheimer's disease.

## 2. Materials and Methods

### 2.1. Participants

In total, 181 native Swiss-German or German speaking adults were recruited in the original Ambizione study at the Memory Clinic FELIX PLATTER, University Department of Geriatric Medicine, Basel, Switzerland. From these, 84 participants, which could be clearly assigned to one group and for which all items produced in the animal fluency task had been thoroughly listed, were included in the study. Forty-two participants (21 male; mean age = 74.4 years; SD = 7.3 years) belonged to the control group and were confirmed cognitively healthy through medical screening and administering thorough neuropsychological testing. In the patient group, there were 42 participants (20 male; mean age = 74.3 years; SD = 6.8 years), of which 24 had been diagnosed with aMCI due to Alzheimer's disease (Albert et al., 2011) according to DSM-IV (American Psychiatric Association, 1994) and Winblad et al. (2004) criteria. Eighteen participants were diagnosed with very early AD according to DSM-IV (American Psychiatric Association, 1994) and NINCDS-ADRDA (McKhann et al., 2011) criteria. The consensus diagnoses were obtained by an interdisciplinary team of experienced clinicians. Demographic information of the different groups can be found in Table 1. Since the three groups differed in age and there is evidence that age affects the performance in the semantic fluency task (Troyer et al., 1997), we used age as a covariate in later analyses. As expected, the participants differed in the Mini Mental State Examination (MMSE) scores but aMCI patients as well as patients with AD scored very high points in the test (see Table 1), which indicates a very early stage of Alzheimer's disease. Written informed consent was obtained from all individuals prior to participating in the study. The study was approved by the local ethical committee and conducted in compliance with all applicable laws and institutional guidelines.

**Table 1:** Demographic information.

	**Healthy (*n* = 42)**	**aMCI (*n* = 24)**	**AD (*n* = 18)**	**χ^2^**	***p***
Sex	21 males	10 males	10 males	0.84	0.657
**Variable**	**Mean (SD)**			***F***	
Education (yrs.)	12.86 (3.14)	13.08 (3.16)	12.22 (3.14)	0.41	0.667
Age (yrs.)	74.38 (7.32)	71.34 (6.59)	78.32 (4.76)	5.68	0.005
MMSE	29.31 (1.00)	28.67 (1.46)	26.61 (1.79)	25.83	<0.001

*aMCI, amnestic Mild Cognitive Impairment; AD, Alzheimer's dementia; MMSE, mini-mental state examination*.

### 2.2. Semantic Fluency Task

In the semantic fluency task, participants were asked to produce as many items within a certain semantic category as they could within 1 min. The item categories were animals, fruits, tools, and vehicles. In this study, we focus on the animal category due to difficulties that patients with Alzheimer's disease exhibit when distinguishing living things in particular (Taylor et al., 2007; Krumm et al., 2021). As proposed by Troyer et al. (1997), we utilized all words produced in the task in the further analyses and did not differentiate between correct and non-correct words (i.e., repetitions or perseverations). In the task, participants produced 224 unique animals altogether, of which the semantic space was formed.

### 2.3. Semantic Distances From Corpus Data

We formulated the semantic space from the 224 unique animals produced in the semantic fluency task using a text corpus, i.e., the 3B-token Google News dataset (Mikolov et al., 2013). The words were first translated from Swiss-German to English. We used a pre-trained word2vec skip-gram model (not yet available in Swiss-German) to find vector representations that predict surrounding words of the given item in a sentence. With this method, 300-dimensional vector representations of words were comprised from unstructured text data, that is, corpus (Mikolov et al., 2013). The code can be found online at https://code.google.com/archive/p/word2vec.

The semantic distances between words were estimated as cosine distances between the word vector representations. Each row in the resulting 224-dimensional semantic dissimilarity matrix described how semantically similar an item is to the other 223 items (within the animal category that we focused on), estimated from zero to one, where values close to zero indicate very similar representations and values close to one very distant representations.

### 2.4. Dimensionality Reduction

To reduce the 224-dimensional matrix description of semantic distances to a tangible visualization, we chose an unsupervised, non-linear dimensionality reduction technique known as t-SNE (van der Maaten and Hinton, 2008). It is designed to visualize the structure of high-dimensional (HD) data with low-dimensional (LD) maps such as two-dimensional scatter plots (van der Maaten and Hinton, 2008). Importantly, for the aim of our present work, t-SNE retains local structures of the data by preserving the distances between points and their nearest neighbors from the original HD data to the LD map. This is done by plotting Gaussian distributions for each point in the HD data and measuring the density of the other points under the Gaussian. The acquired probability functions are compared to similarly acquired t-distributed similarity functions in the LD data and are measured by the Kullback-Leibler divergence, which t-SNE tries to minimize. Student's t-distribution is used because it allows for better modeling of far apart distances, since it does not give as much emphasis on values at the extreme ends of the distribution (van der Maaten and Hinton, 2008).

As suggested by van der Maaten and Hinton (2008), prior to t-SNE we applied another dimensionality reduction technique (MDS; Buja et al., 2008) to reduce the number of dimensions. MDS reduced the number of the dimensions from 224 to 50 (as suggested by van der Maaten and Hinton, 2008) which was the number of dimensions that explained 96% of the variance in the data. Finally, t-SNE was implemented on these 50 dimensions, which resulted in a two-dimensional plot. After visual inspection of the sub-category borders in the plot, we divided the 224 unique items into sub-categories, so that each item belonged to one sub-category. The item labels were visible during the labeling process.

The dimensionality reduction model was executed with Python 3.7 (Van Rossum and Drake, 2009) using the package sklearn.manifold (Pedregosa et al., 2011). Multiple model solutions with different perplexity parameter values were executed. The perplexity parameter defines the number of points falling under the probability distribution, thus perplexity can be considered to set the number of effective nearest neighbors estimated for each point, and is suggested to be between 5 and 50 in a t-SNE model (van der Maaten and Hinton, 2008). As we were interested in the local clusters, we used a perplexity value of 20 in the final model. However, different perplexity values did not greatly affect the overall output of the model, as the manually created sub-category structure remained stable (see Supplementary Material). A number of 1,500 iterations was found to establish a stable model. t-SNE was run multiple times, as suggested by van der Maaten and Hinton (2008), to achieve the lowest Kullback-Leibler divergence, which was 0.88 in the final model.

### 2.5. Statistical Analyses

Statistical analyses were executed with IBM SPSS Statistics (Version 25). To examine the participants' performance in the semantic fluency task, we calculated the sum of items in each sub-category per participant (i.e., how many pets or birds the participant produced) and summed all these items to get the total number of words produced in the task (“Number of words”). We also examined the number of words in each sub-category and divided it by the number of words produced in the task to get proportional information of each sub-category. In addition, we recorded the number of sub-categories visited in the task (“Sub-categories”). We defined switching (“Switching”) as moving from one sub-category to another, calculated the sum of switching for each participant and divided that by the number of words each participant produced (“Adjusted switching”). As we sought to examine movement in the semantic space as thoroughly as possible, we also chose to inspect a novel variable that would capture not only unidirectional movement from cluster to cluster but also describe revisiting previously utilized areas in the semantic space. For this purpose, we examined the number of times a participant returned to a sub-category which they had previously visited (“Returns”) and adjusted that number with the total number of words produced (“Adjusted returns”). To further study how returning to a sub-category affected the number of words within a sub-category, we also inspected the average pattern of visitations to a sub-category for each group and how many of them were returns.

Normality of the data was evaluated with Q-Q plots. The effect of belonging to a diagnostic group on the number of words produced in the task was examined with one-way analysis of variance (ANOVA). To examine the effects of belonging to a diagnostic group (healthy, aMCI, or AD) on moving in the semantic space, sub-categories, switching, adjusted switching, and adjusted returns were used as dependent variables in one-way multivariate analysis of variance (MANOVA), which was conducted to minimize the likelihood of type 1 error. Separate linear multinomial logistic regression models were used to predict diagnostic group with the number of categories, adjusted switching, and adjusted returns as independent variables. Since there is evidence that age affects the performance in the semantic fluency task (Troyer et al., 1997), we included age as a control variable in each model, in addition to the number of words. We did not combine the independent variables into one model, due to multicollinearity issues between some variables.

## 3. Results

### 3.1. Corpus Data

**Figure 1:**
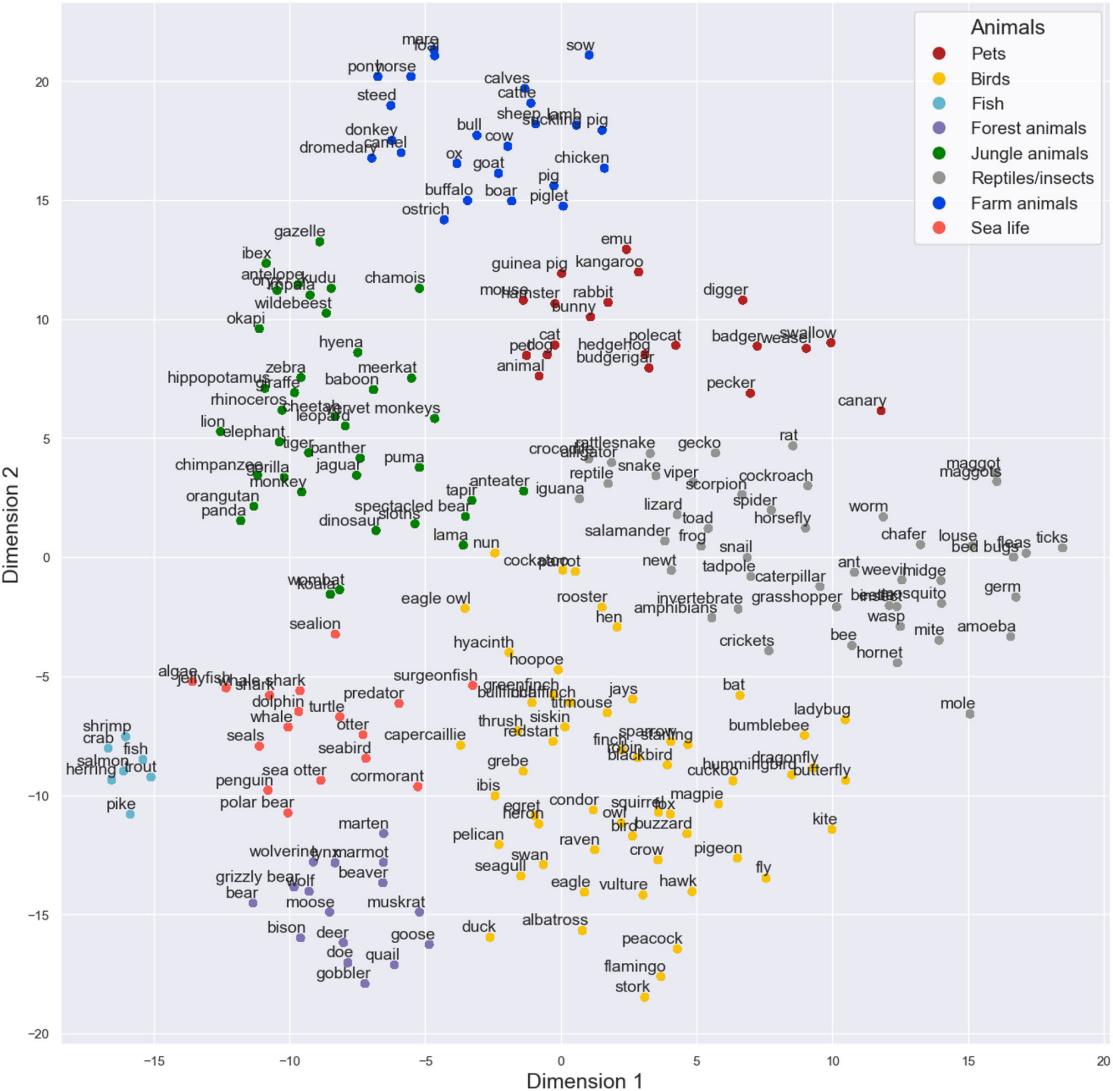
Two-dimensional visualization of the animals produced in the semantic fluency task by the t-SNE model on the 50 dimensions of multidimensional scaling. In the figure, different sub-category labels are presented based on visual inspection as different colors. t-SNE produced some very tight categories (such as sealife in the left bottom corner) and some sub-categories that are more loose (such as birds in the bottom center).

The results from the t-SNE analysis of the corpus data based on the 50 components produced by MDS can be seen in Figure 1. After visual inspection, we formed eight sub-categories based on the t-SNE result. The clusters could be labeled as pets, birds, fish, forest animals, jungle animals, reptiles and insects, farm animals, and sea life. The sub-categories were used in the subsequent analyses. Further, the visually inspected sub-category structure was strongly supported by also the k-means clustering solution (see Supplementary Figure 5).

### 3.2. Behavioral Data

**Figure 2:**
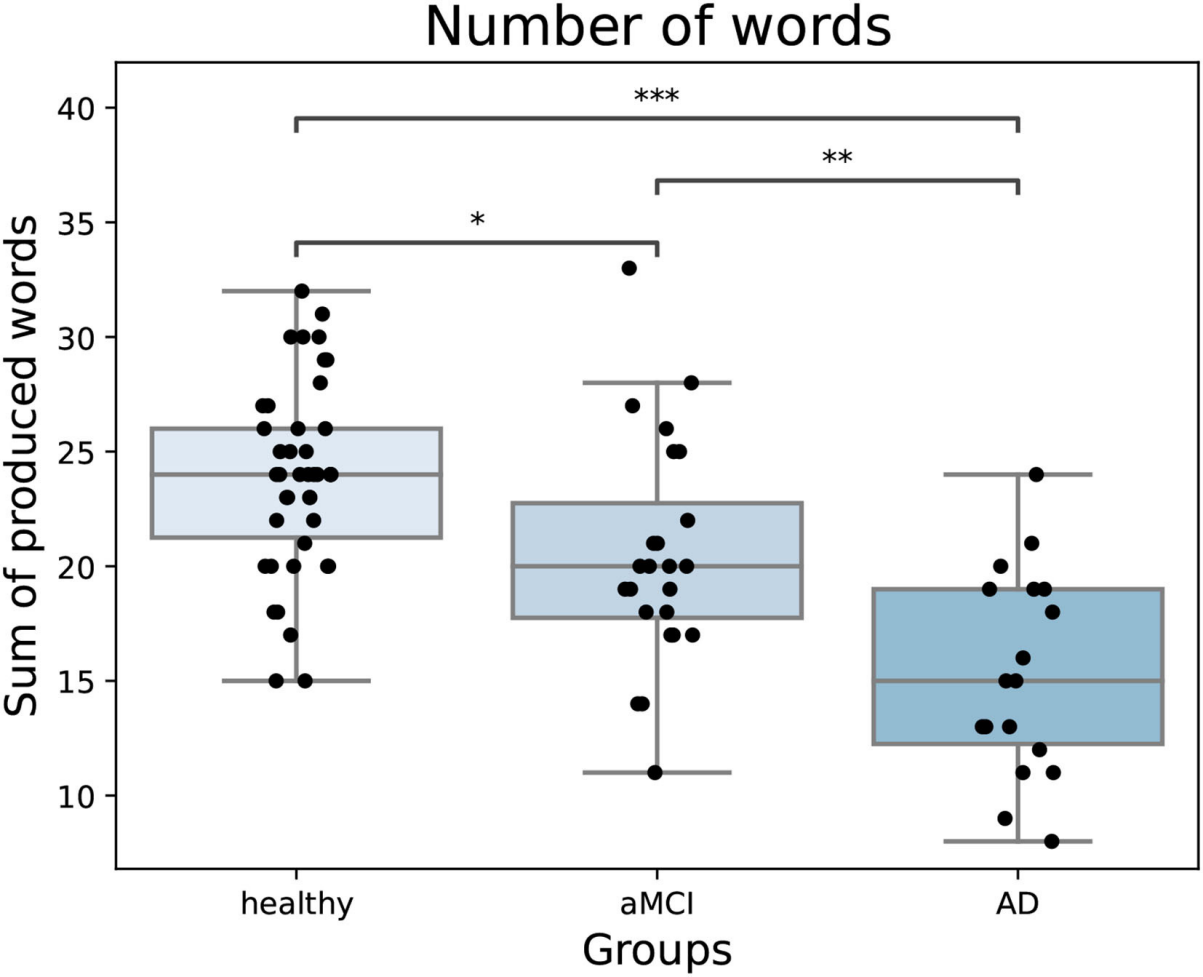
The number of words produced in the task by groups. Healthy participants name more words compared to both patient groups and aMCI patients name more compared to AD patients. In the boxplot, minimum, first quartile, median, third quartile, and maximum are shown. Individual data points are shown as black dots. aMCI, amnestic Mild Cognitive Impairment; AD, Alzheimer's dementia. *p < 0.05, **p < 0.01, ***p < 0.001.

Overall, the semantic fluency variables were strongly correlated with each other (Table 2). After adjusting, switching was no longer correlated with the number of words but adjusted returns had a small positive correlation with the number of words. There was a significant difference between groups in the number of words produced [*F*_(2,81)_ = 23.49, *p* < 0.001, partial η^2^ = 0.37]. As expected, healthy participants named more animals compared to both aMCI and AD patients, and aMCI patients named more compared to AD patients (Figure 2). Overall, farm animals, jungle animals, pets, and birds were the most often named sub-categories. There were many participants in all groups that named multiple birds in the task. Fish and sea life were sub-categories that were used less often, as many participants named zero to one items from these sub-categories.

**Table 2:** Spearman correlations between semantic fluency variables.

**Variables**	**1**	**2**	**3**	**4**	**5**
1. Number of words					
2. Sub-categories named	0.46^***^				
3. Switches	0.65^***^	0.68^***^			
4. Adjusted switches	−0.01	0.44^***^	0.61^***^		
5. Returns	0.59^***^	0.39^**^	0.93^***^	0.59^**^	
6. Adjusted returns	0.24^*^	0.25^*^	0.78^***^	0.80^***^	0.90^***^

* *p < 0.05*,

** *p < 0.01*,

*** *p < 0.001*.

**Figure 3:**
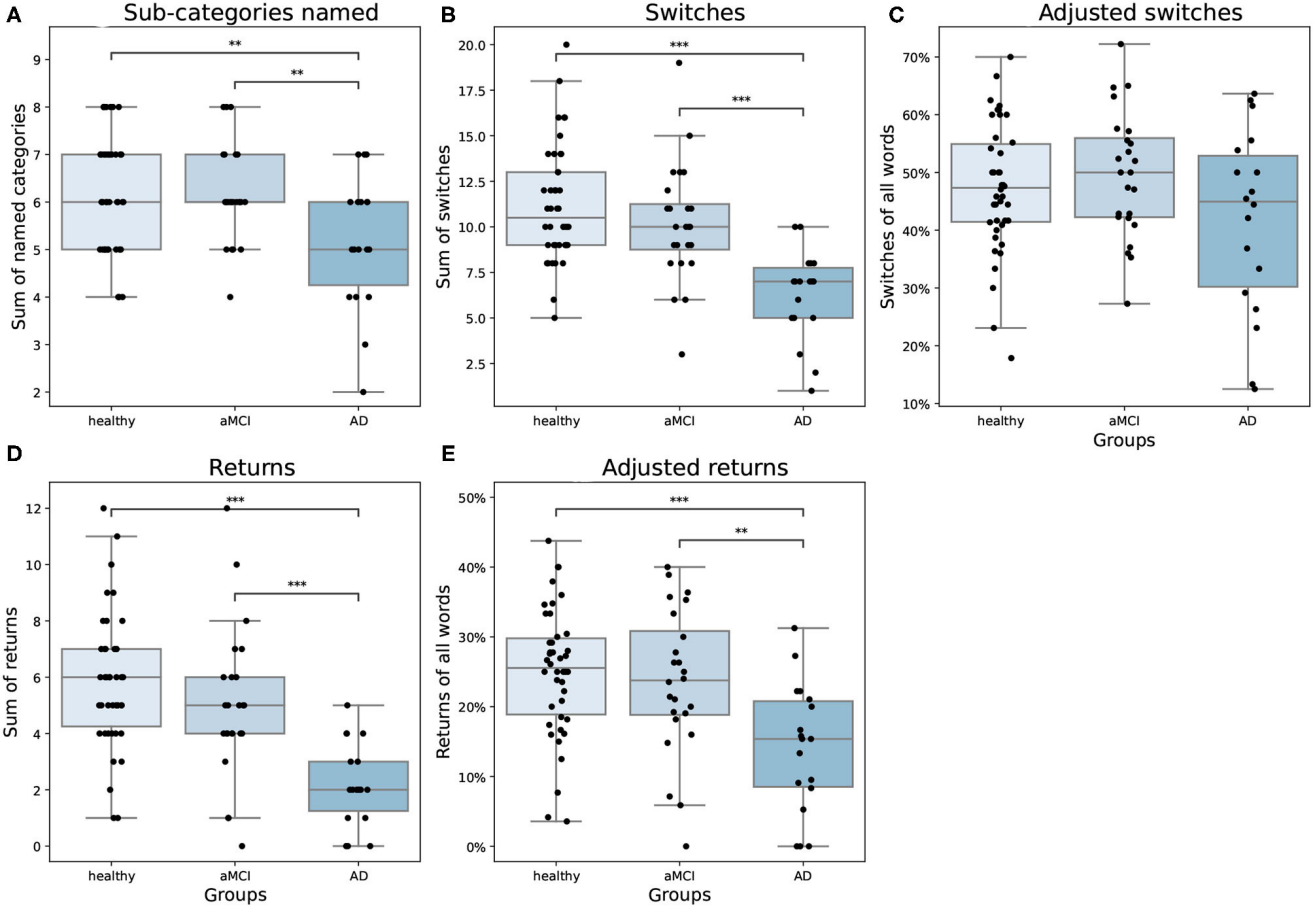
**(A)** Number of sub-categories named, **(B)** switches, **(C)** adjusted switches, **(D)** returns, and **(E)** adjusted returns as means and standard errors by groups. In the boxplots, minimum, first quartile, median, third quartile, and maximum are shown. Individual data points are shown as black dots. aMCI, amnestic Mild Cognitive Impairment; AD, Alzheimer's dementia. **p < 0.01, ***p < 0.001.

There were differences between groups in how they moved in the semantic space [*F*_(8,156)_ = 6.31, *p* < 0.001, Wilk's Λ = 0.57, partial η^2^ = 0.25]. The groups visited different numbers of sub-categories during the task [*F*_(2,81)_ = 5.45, *p* = 0.006, partial η^2^ = 0.12]. AD patients named fewer sub-categories compared to both aMCI patients and healthy controls (Figure 3A). In our data, no AD patient visited all of the eight sub-categories. Consistent with previous literature, the groups also differed from each other in switching from a sub-category to another [*F*_(2,81)_ = 16.80, *p* = < 0.001, partial η^2^ = 0.29], where the AD group switched sub-categories less often than aMCI patients and healthy controls (Figure 3B). When the number of switching was adjusted with the overall number of words produced, there was no statistically significant difference between groups [*F*_(2,81)_ = 2.24, *p* = 0.113, partial η^2^ = 0.05; Figure 3C]. However, the adjusted returns variable revealed a highly significant difference between the groups [*F*_(2,81)_ = 8.69, *p* < 0.001, partial η^2^ = 0.18]. In the pairwise analyses, we found that both healthy participants and aMCI patients returned to the sub-categories they had previously visited more often compared to AD patients (Figure 3D), even when the number of words produced was controlled (Figure 3E). On average, AD patients returned to a previously used category 2.17 times (SD = 1.47), aMCI patients 5.04 times (SD = 2.71), and healthy controls 5.98 times (SD = 2.67).

**Figure 4:**
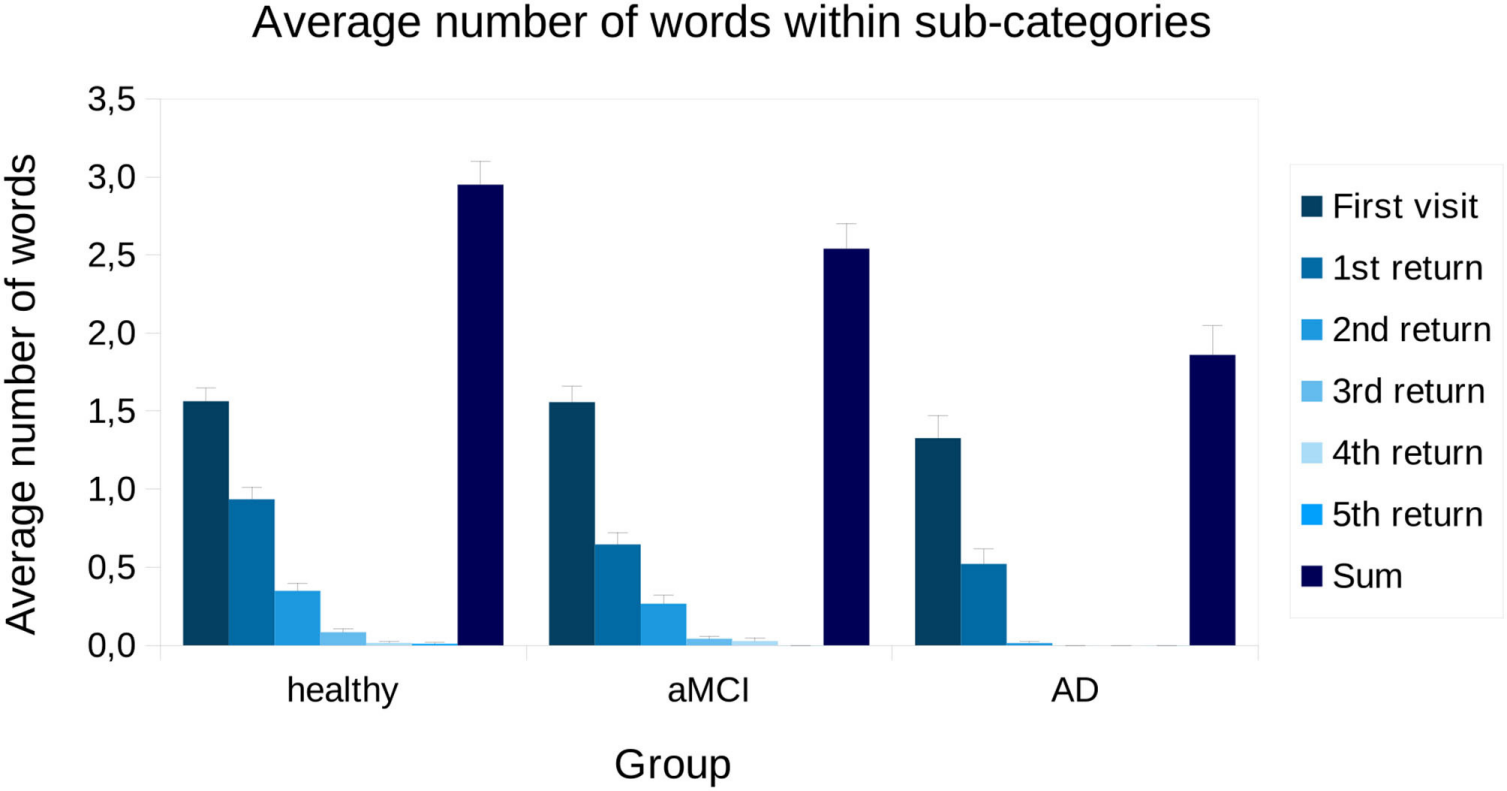
Visitations and returns presented for each group across all sub-categories. Bars represent standard error. aMCI, amnestic Mild Cognitive Impairment; AD, Alzheimer's dementia; Sum, average sum of words produced within a sub-category.

To inspect returning to a sub-category more closely, the average number of words named in each visitation to a sub-category is presented in Figure 4. From one-way ANOVA, we found that, on average, groups differ in how many words were named in each sub-category [*F*_(2,669)_ = 9.57, *p* < 0.001]. In the Bonferroni corrected, bootstrapped pairwise analyses, we found that AD patients produced fewer words compared to aMCI patients (*p* < 0.05) and healthy controls (*p* < 0.001). The average number of words named on the first visit to the sub-group was not statistically different between groups [*F*_(2,669)_ = 1.28, *p* = 0.28]. Statistical comparisons between subjects on 4 and 5 returns were not made, since there were only a few participants who made that many returns (made by four healthy and two aMCI participants, and two healthy participants, respectively). Further, as no AD patients revisited sub-categories more than 2 times, we only analyzed returns 1 and 2. For 1 and 2 returns, there were statistical differences between groups [*F*_(2,669)_ = 6.43, *p* < 0.01; *F*_(2,669)_ = 9.56, *p* < 0.001]. In the pairwise analyses, we found that during the first return AD patients named fewer words compared to healthy controls (*p* < 0.01). Further, aMCI patients produced fewer words compared to healthy controls (*p* < 0.05). During the second return, AD patients produced fewer words compared to both healthy participants (*p* < 0.001) and aMCI patients (*p* < 0.01).

The results from the multinomial logistic models are presented in Table 3. Model 1, which consisted of only the number of words and age, was statistically significant and explained half of the variation in the data. To Models 2 and 3 we added the adjusted switching and the number of sub-categories, respectively. Even though both models themselves were statistically significant, neither the adjusted switching nor the number of sub-categories were statistically significant. Therefore, these variables did not improve the models' fit to the data. Neither adjusted switching nor the number of sub-categories had a significant, independent effect in the models when the number of words and age were controlled. In Model 4, adjusted returns was a statistically significant predictor even when the number of words and age were controlled. Adding adjusted returns into Model 1 improved the explanatory power by six percent. However, the Bayesian information criterion (BIC) and Akaike's information criterion (AIC) indicators for model fit are not convergent, as the marginally higher BIC-index (compared to Model 1) implicates worse fit.

**Table 3:** Multinomial logistic regression models.

**Model**	** ${\tilde{\chi }}^{2}$ **	**Pseudo *R*^2^^+^**	**BIC**	**AIC**	***p***
**Model 1**	46.74	0.49	153.65	139.07	<0.001
Number of words	35.94				<0.001
Age	9.24				0.010
**Model 2**	51.74	0.53	157.52	138.07	<0.001
Number of words	38.77				<0.001
Age	8.37				0.015
Adjusted switches	5.00				0.082
**Model 3**	47.23	0.49	162.03	142.58	<0.001
Number of words	29.34				<0.001
Age	8.33				0.016
Sub-categories	0.49				0.783
**Model 4**	54.78	0.55	154.48	135.04	<0.001
Number of words	30.49				<0.001
Age	10.27				0.006
Adjusted returns	8.03				0.018

+ *Nagelkerke's Pseudo R^2^ is used. BIC, Bayesian information criterion; AIC, Akaike information criterion*.

Pairwise comparisons between groups from Model 4 are depicted in Table 4. The number of words differentiated AD patients from both aMCI patients and healthy controls. When the number of words increased by one, the odds ratio for healthy controls and aMCI grew by 1.54 and 1.27, respectively, compared to the AD group. Age differentiated aMCI patients from AD, so that when age increased by one year, the odds ratio for being in the AD group grew by 1.27. Finally, the adjusted returns differentiated healthy and aMCI participants from AD patients. When adjusted returns increased by one percent, the odds ratio for healthy controls and aMCI patients grew by 1.13 and 1.10, respectively, compared to the AD group.

**Table 4:** Model 4: Multinomial logistic regression analysis on belonging to a group with adjusted returns, the number of words, and age as independent variables.

	**Healthy vs. AD**						**aMCI vs. AD**					
	**B**	**S.E**	**Wald**	**Exp(B)**	**95% C.I**		**B**	**S.E**	**Wald**	**Exp(B)**	**95% C.I**	
					**L**	**U**					**L**	**U**
Intercept	−4.38	5.01	0.76				6.98	4.70	2.21			
Adjusted returns	0.12	0.05	6.72^**^	1.13	1.03	1.23	0.09	0.05	4.24^*^	1.10	1.00	1.20
Number of words	0.43	0.11	16.78^***^	1.54	1.25	1.90	0.24	0.19	5.86^*^	1.27	1.54	1.91
Age	−0.07	0.06	1.47	0.93	0.82	1.05	−0.17	0.06	7.25^**^	1.10	1.00	1.20

*Reference category is the AD group. Adjusted returns variable is multiplied by hundred for easier interpretation. aMCI, amnestic Mild Cognitive Impairment; AD, Alzheimer's dementia; B, unstandardized beta co-efficient; S.E, standard error; Exp(B), odds ratio; C.I, confidence interval; L, lower bound; U, upper bound*.

* *p < 0.05*,

** *p < 0.01*,

*** *p < 0.001*.

Finally, we inspected the classification rates of the Models 1 and 4 (Table 5). Adding the adjusted returns to Model 1 did not improve the overall classification power of the model (one percent), but it did improve the classification of AD patients by 16%. However, Model 4 was not as accurate as Model 1 in classifying healthy controls (i.e., Model 4 classified more healthy controls to the aMCI patient category). Yet, Model 1 mistook more AD patients as healthy controls compared to Model 4. The AD patients were overall better classified in Model 4 compared to Model 1. Neither of the models was able to classify aMCI patients above the 0.5 random cut-off point.

**Table 5:** Classification amounts and rates (%) for Models 1 and 4 presented as a confusion matrix.

**Model 1**					**Model 4**				
	**Predicted**					**Predicted**			
**Observed**	**Healthy**	**aMCI**	**AD**	**Correct (%)**	**Observed**	**Healthy**	**aMCI**	**AD**	**Correct (%)**
Healthy	33	7	2	78.6	Healthy	31	10	1	73.8
aMCI	9	10	5	41.7	aMCI	11	10	3	41.7
AD	7	0	11	61.1	AD	4	0	14	77.8
Overall (%)	58.3	20.2	21.4	64.3	Overall (%)	54.8	23.8	21.4	65.5

*aMCI, amnestic Mild Cognitive Impairment; AD, Alzheimer's dementia*.

## 4. Discussion

As a novel finding, the results from the present study suggest that AD patients return less often to already visited sub-categories compared to both aMCI patients and healthy participants. Furthermore, we found evidence that there are no differences between groups in how many words are produced for the first time visiting a sub-category, but rather the differences emerge from how many times the participants return to the sub-categories and how many items they produce within each visit. These results may suggest that especially AD patients lack the ability to utilize the semantic space by flexibly changing strategy even though they are able to produce a similar amount of words compared to normal controls when visiting a sub-category for the first time. Further, this decline is also already visible in aMCI patients, which is considered the prodromal stage of AD.

In discriminating between groups, we found that only the adjusted returns provided further information in addition to the number of words produced and age (Table 3). Adding adjusted returns to the model marginally enhanced the classification of the whole model (Model 4) and seemed to do better in discriminating AD patients from healthy participants. However, we did not utilize a cross-validation procedure in creating the regression model, which is a limitation considering our relatively small sample size per each group. Further, the classification rates of Model 4 for healthy participants were slightly worse compared to Model 1. Since the behavior of aMCI patients in the semantic fluency task seemed more similar to healthy controls (see Figure 3E), we also combined these groups and compared them to the AD group in additional analyses (see Supplementary Material). Even after combining the healthy controls with the aMCI group, returning to a sub-category was helpful in discriminating the AD patients. These results support the idea that evaluating returns to a sub-category may be of aid in discriminating between AD patients and healthy controls. Overall, the use of adjusted returns may aid clinicians in diagnosing early AD, but more research is needed to replicate our findings.

In line with previous studies, healthy participants produced more words in the semantic fluency task than aMCI and AD patients (Troyer et al., 1998; Fagundo et al., 2008; Raoux et al., 2008; Price et al., 2012). Furthermore, healthy participants produced more sub-categories and performed more switching compared to AD patients. aMCI patients also performed better than AD patients in producing more words, sub-categories, and switching. However, when we inspected the adjusted switching variable, there were no statistically significant differences between groups in switching. Adjusted switching and sub-categories did not bring additional information in classifying participant groups. These findings suggest that in addition to the number of words produced in the semantic fluency task, the number of sub-categories and the adjusted number of switching do not seem to provide additional information in discriminating AD and aMCI patients from healthy controls.

Based on the feature-vector representations from the corpus data, we were able to visualize the semantic space in a two-dimensional space with the t-SNE dimensionality reduction algorithm and form sub-categories of the animal category. The results of the t-SNE model were stable and did not change drastically with different perplexity parameters (see Supplementary Material). Moreover, a reliable sub-category structure could also be created with an alternative method, the k-means clustering (see Supplementary Figure 5). These results speak for the usefulness of corpus based methods, combined with dimensionality reduction methods in describing structures within the semantic space.

Since the sub-categories created were based on the statistical properties of condensed feature vectors of words, they were not purely biological sub-categories. For instance, words “fox” and “squirrel” were labeled as birds since they were closely located to that sub-category in the final model. This labeling is supported also by the other models in the Supplementary Material, as these words seem to flock to the bird cluster generally. These results can be explained by their closeness to words “owl” and “crow” as these animals also often occur together in fairy tales, for example. There were some other outliers that did not clearly belong to one specific sub-category but were always situated near them. For instance, “swallow,” “pecker,” “weasel,” and “badger” seemed to cluster together and close to pets in all of the models, so they were put into the pet sub-category in the final model. It seems that the word2vec algorithm is able to capture the multidimensional nature of the semantic space from the corpus data. Further, t-SNE worked very effectively on the cosine distance data in classifying high-dimensional items, as previously suggested by other authors (van der Maaten and Hinton, 2008). Combining these methods seems to help us capture multidimensional information very well. Finally, t-SNE can be considered an essential visual aid for performing the manual evaluation of sub-categories based on the corpus derived data.

Feature-based vector modeling combined with t-SNE visualization helps us to achieve a simple, condense visualization of the semantic space, based on which an unambiguous sub-category structure can be formed with visual inspection. Further, using the vector model and t-SNE mapping, we are able to visualize the semantic space, which helps us to form an unambiguous sub-category solution for the data of the specific task for all participants. These methods therefore allow us to perform between-individual comparisons on switching and the number of items in the sub-categories. Without a clear sub-category structure, comparing returning to a sub-category between individuals would be meaningless. Vector modeling combined with dimensionality reduction thus enables us to create a two-dimensional semantic space with which to quantify returning to a sub-category. According to the neurocognitive framework, the patterns of networks responsible for feature activation in the brain can be considered relatively stable (McRae et al., 1997; Vigliocco et al., 2004). Thus, we may assume that semantic space and the sub-categories within are also relatively stable across individuals. This supports the idea of a universal semantic mapping.

In this article, we created a single semantic structure based on an English corpus after we translated the words from Swiss-German. For future research, it may be fruitful to further investigate and visualize the differences between corpus data from other languages (when possible) to examine whether there are differences in the semantic space structure of different languages. Further, it would be important to replicate our findings in other categories of the semantic task (fruits, vegetables, tools, and vehicles). Moreover, it would be important to study, using the method presented in this article, whether the overall sub-category structures of semantic spaces differ between healthy controls and patients with prodromal and early Alzheimer's disease.

In the present study, we aimed to zoom inside the sub-categories of the semantic fluency task, which enabled us to find a new variable for describing moving in the semantic space. The results of this study demonstrate that in addition to classic higher-level categories, such as living vs. non-living or animals, fruits, vehicles, and tools, it is also possible to examine the sub-categories within the category. In the present study, this was executed using an internet-based corpus, word2vec skip-gram model, and a dimensionality reduction algorithm. These results give new insights by zooming inside the higher-level categories and, thus, we suggest that these methods may be useful in gaining more knowledge on how individuals utilize the semantic space such as in the semantic fluency task. Further, these methods are easily scalable for even larger vocabularies and can be easily reused for new data sets. For future research, we suggest that examining semantic distances in differentiating between individuals may prove useful.

### 4.1. Limitations

Our methodology has some limitations. Overall, using a dimensionality reduction method on high-dimensional data leads to loss of information. Further, t-SNE has some limitations as a dimensionality reduction and a classification method. Since t-SNE is data-driven, its results may not identically replicate in other data sets. In this study, we aimed to improve replicability by limiting the number of sub-categories. This approach has the drawback of possibly losing information on individual categorization strategies. For instance, in our data, “mouse” and “rat” belong to different sub-categories (pets and reptiles/insects, respectively), which could also be categorized to the same sub-category using another logic. However, we propose that the general tendency with reduced sub-category returns can be demonstrated regardless of differences in clustering approaches and parameters. Another limitation is that the sub-categories were formed on visual inspection based on the t-SNE visualization, as it is not advisable to use an actual clustering algorithm on the t-SNE results because t-SNE does not preserve distances between sub-categories or alternatively regards them meaningless (van der Maaten and Hinton, 2008). Further, as the item labels were not hidden when performing the manual clustering, semantic knowledge of the authors may have affected the evaluation of sub-category borders. However, we suggest that since the t-SNE results were relatively stable across models, and the k-means clustering results mostly corresponded with the manually labeled sub-categories, visual inspection of the data was sufficient to divide the semantic space into meaningful sub-categories. Finally, as we have performed the behavioral analyses using only the sub-categories achieved with non-blind visual inspection of the t-SNE solution, it remains to be empirically tested whether other clustering methods used on semantic fluency task data replicate the behavioral findings. The present results also do not address the question on how the clusters derived using word2vec and t-SNE compare to alternative methods, and, e.g., fully manual labeling. This methodological comparison remains to be addressed in future studies.

### 4.2. Conclusions

In the present study, we aimed to utilize a new method for emulating the process of moving within the semantic space. Based on our results, a corpus derived feature-vector model visualized with t-SNE provides a valuable tool for understanding the semantic space and its sub-categories, which individuals seem to utilize efficiently in the semantic fluency task. Using this tool, we found that in the fluency task, inspecting returns to a sub-category may yield additional information for differentiating patients with AD from cognitively healthy controls and, thus, may be useful for clinicians when diagnosing early Alzheimer's disease. However, number of sub-categories and switching did not substantially improve differentiation between patients and healthy controls. We hope that these results offer helpful insight for clinicians to understand the behavior of prodromal and very early Alzheimer's disease patients in the semantic fluency task and promote discovery of these diseases at the most initial stage possible.

## Data Availability Statement

The original contributions presented in the study are included in the article/Supplementary Material, further inquiries can be directed to the corresponding author/s.

## Ethics Statement

The studies involving human participants were reviewed and approved by Ethikkommission Nordwest–und Zentralschweiz (EKNZ) Hebelstrasse 53 CH-4056 Basel Switzerland. The patients/participants provided their written informed consent to participate in this study.

## Author Contributions

AS, SKi, and SKr contributed to conception and design of the study. AS organized the database, performed the statistical analysis, and wrote the first draft of the manuscript. AS, SKi, RS, and SKr wrote sections of the manuscript. All authors contributed to manuscript revision and read and approved the submitted version.

## Funding

This study was supported by a grant from the Swiss National Science Foundation (Ambizione fellowship PZ00P1_126493) awarded to Kirsten I. Taylor, Ph.D. (see section Acknowledgements), Academy of Finland Research Grants #286070 to SKi and #315553 to RS, and Sigrid Jusélius Foundation grant (no grant number) to RS.

## Conflict of Interest

The authors declare that the research was conducted in the absence of any commercial or financial relationships that could be construed as a potential conflict of interest.

## Publisher's Note

All claims expressed in this article are solely those of the authors and do not necessarily represent those of their affiliated organizations, or those of the publisher, the editors and the reviewers. Any product that may be evaluated in this article, or claim that may be made by its manufacturer, is not guaranteed or endorsed by the publisher.

## References

[B1] Abwender D. A. Swan J. G. Bowerman J. T. Connolly S. W. (2001). Qualitative analysis of verbal fluency output: review and comparison of several scoring methods. Assessment 8, 323–338. 10.1177/107319110100800308 11575625

[B2] Albert M. S. DeKosky S. T. Dickson D. Dubois B. Feldman H. H. Fox N. C.. (2011). The diagnosis of mild cognitive impairment due to Alzheimer's disease: recommendations from the National Institute on Aging-Alzheimer's Association workgroups on diagnostic guidelines for Alzheimer's disease. Alzheimer's Dement. 7, 270–279. 10.1016/j.jalz.2011.03.008 21514249 PMC3312027

[B3] American Psychiatric Association (1994). Diagnostic and Statistical Manual of Mental Disorders. Washington, DC: American Psychiatric Association Press.

[B4] Amieva H. Le Goff M. Millet X. Orgogozo J. M. Pérès K. Barberger-Gateau P.. (2008). Prodromal Alzheimer's disease: successive emergence of the clinical symptoms. Ann. Neurol. 64, 492–498. 10.1002/ana.21509 19067364

[B5] Buja A. Swayne D. F. Littman M. L. Dean N. Hofmann H. Chen L. (2008). Data visualization with multidimensional scaling. J. Comput. Graph. Stat. 17, 444–472. 10.1198/106186008X318440

[B6] Clarke A. Tyler L. K. (2014). Object-specific semantic coding in human perirhinal cortex. J. Neurosci. 34, 4766–4775. 10.1523/JNEUROSCI.2828-13.2014 24695697 PMC6802719

[B7] DeLise T. (2021). Data segmentation via t-SNE, DBSCAN, and random forest. arXiv:2010.13682. 10.1007/978-3-030-80126-7_11

[B8] Devassy B. George S. Nussbaum P. (2020). Unsupervised clustering of hyperspectral paper data using t-SNE. J. Imaging 6:29. 10.3390/jimaging6050029 34460731 PMC8321027

[B9] Ellis A. W. Young A. W. (2013). Human Cognitive Neuropsychology: A Textbook With Readings. New York, NY: Psychology Press. 10.4324/9780203727041

[B10] Epker M. O. Lacritz L. H. Munro Cullum C. (1999). Comparative analysis of qualitative verbal fluency performance in normal elderly and demented populations. J. Clin. Exp. Neuropsychol. 21, 425–434. 10.1076/jcen.21.4.425.890 10550803

[B11] Fagundo A. B. López S. Romero M. Guarch J. Marcos T. Salamero M. (2008). Clustering and switching in semantic fluency: predictors of the development of Alzheimer's disease. Int. J. Geriatr. Psychiatry 23, 1007–1013. 10.1002/gps.2025 18416452

[B12] Henry J. D. Crawford J. R. Phillips L. H. (2004). Verbal fluency performance in dementia of the Alzheimer's type: a meta-analysis. Neuropsychologia 42, 1212–1222. 10.1016/j.neuropsychologia.2004.02.001 15178173

[B13] Kivisaari S. L. Tyler L. K. Monsch A. U. Taylor K. I. (2012). Medial perirhinal cortex disambiguates confusable objects. Brain 135, 3757–3769. 10.1093/brain/aws277 23250887 PMC3525054

[B14] Kivisaari S. L. van Vliet M. Hultén A. Lindh-Knuutila T. Faisal A. Salmelin R. (2019). Reconstructing meaning from bits of information. Nat. Commun. 10, 1–11. 10.1038/s41467-019-08848-0 30804334 PMC6389990

[B15] Koo T. K. Li M. Y. (2016). A guideline of selecting and reporting intraclass correlation coefficients for reliability research. J. Chiropract. Med. 15, 155–163. 10.1016/j.jcm.2016.02.012 27330520 PMC4913118

[B16] Krumm S. Berres M. Kivisaari S. L. Monsch A. U. Reinhardt J. Blatow M.. (2021). Cats and apples: semantic fluency performance for living things identifies patients with very early Alzheimer's disease. Arch. Clin. Neuropsychol. 36, 838–843. 10.1093/arclin/acaa109 33237317 PMC8292926

[B17] Lezak M. D. Howieson D. B. Loring D. W. Fischer J. S. (2004). Neuropsychological Assessment. Oxford, MI: Oxford University Press.

[B18] March E. Pattison P. (2006). Semantic verbal fluency in Alzheimer's disease: approaches beyond the traditional scoring system. J. Clin. Exp. Neuropsychol. 28, 549–566. 10.1080/13803390590949502 16624783

[B19] McKhann G. M. Knopman D. S. Chertkow H. Hyman B. T. Jack C. R. Kawas C. H.. (2011). The diagnosis of dementia due to Alzheimer's disease: recommendations from the National Institute on Aging-Alzheimer's Association workgroups on diagnostic guidelines for Alzheimer's disease. Alzheimer's Dement. 7, 263–269. 10.1016/j.jalz.2011.03.005 21514250 PMC3312024

[B20] McRae K. De Sa V. R. Seidenberg M. S. (1997). On the nature and scope of featural representations of word meaning. J. Exp. Psychol. Gen. 126, 99–130. 10.1037/0096-3445.126.2.99 9163932

[B21] Mikolov T. Sutskever I. Chen K. Corrado G. S. Dean J. (2013). Distributed representations of words and phrases and their compositionality. Adv. Neural Inform. Process. Syst. 26, 3111–3119. Available online at: https://arxiv.org/pdf/1310.4546.pdf 31840584

[B22] Mistridis P. Krumm S. Monsch A. U. Berres M. Taylor K. I. (2015). The 12 years preceding mild cognitive impairment due to Alzheimer's disease: the temporal emergence of cognitive decline. J. Alzheimer's Dis. 48, 1095–1107. 10.3233/JAD-150137 26402083 PMC4927842

[B23] Nadkarni P. M. Ohno-Machado L. Chapman W. W. (2011). Natural language processing: an introduction. J. Am. Med. Inform. Assoc. 18, 544–551. 10.1136/amiajnl-2011-000464 21846786 PMC3168328

[B24] Patterson K. Nestor P. J. Rogers T. T. (2007). Where do you know what you know? The representation of semantic knowledge in the human brain. Nat. Rev. Neurosci. 8, 976–987. 10.1038/nrn2277 18026167

[B25] Pedregosa F. Varoquaux G. Gramfort A. Michel V. Thirion B. Grisel O.. (2011). Scikit-learn: machine learning in python. J. Mach. Learn. Res. 12, 2825–2830. Available online at: https://www.jmlr.org/papers/volume12/pedregosa11a/pedregosa11a.pdf?ref=https://githubhelp.com

[B26] Pekkala S. (2004). Semantic fluency in mild and moderate Alzheimer's disease (Doctoral thesis). Department of Phonetics; University of Helsinki, Helsinki, Finland.

[B27] Price S. E. Kinsella G. J. Ong B. Storey E. Mullaly E. Phillips M.. (2012). Semantic verbal fluency strategies in amnestic mild cognitive impairment. Neuropsychology 26, 490–497. 10.1037/a0028567 22746308

[B28] Raoux N. Amieva H. Le Goff M. Auriacombe S. Carcaillon L. Letenneur L.. (2008). Clustering and switching processes in semantic verbal fluency in the course of Alzheimer's disease subjects: results from the PAQUID longitudinal study. Cortex 44, 1188–1196. 10.1016/j.cortex.2007.08.019 18761132

[B29] Saranpää A. M. (2020). Moving in semantic space in prodromal and very early Alzheimer's disease: a characterisation of the semantic fluency task (Master's thesis). Faculty of Medicine; University of Helsinki, Helsinki, Finland.

[B30] Taskesen E. Reinders M. J. T. (2016). 2D representation of transcriptomes by t-SNE exposes relatedness between human tissues. PLoS ONE 11:e0149853. 10.1371/journal.pone.0149853 26906061 PMC4764374

[B31] Taylor K. I. Devereux B. J. Tyler L. K. (2011). Conceptual structure: towards an integrated neurocognitive account. Lang. Cogn. Process. 26, 1368–1401. 10.1080/01690965.2011.568227 23750064 PMC3673226

[B32] Taylor K. I. Moss H. E. Tyler L. K. (2007). The conceptual structure account: a cognitive model of semantic memory and its neural instantiation, in Neural Basis of Semantic Memory, eds Hart J. Kraut M. A. (Cambridge: Cambridge University Press), 265–301. 10.1017/CBO9780511544965.012

[B33] Troyer A. K. Moscovitch M. Winocur G. (1997). Clustering and switching as two components of verbal fluency: evidence from younger and older healthy adults. Neuropsychology 11:138. 10.1037/0894-4105.11.1.138 9055277

[B34] Troyer A. K. Moscovitch M. Winocur G. Leach L. Freedman M. (1998). Clustering and switching on verbal fluency tests in Alzheimer's and Parkinson's disease. J. Int. Neuropsychol. Soc. 4, 137–143. 10.1017/S1355617798001374 9529823

[B35] Tyler L. Moss H. Durrant-Peatfield M. Levy J. (2000). Conceptual structure and the structure of concepts: a distributed account of category-specific deficits. Brain Lang. 75, 195–231. 10.1006/brln.2000.2353 11049666

[B36] Tyler L. K. Chiu S. Zhuang J. Randall B. Devereux B. J. Wright P.. (2013). Objects and categories: feature statistics and object processing in the ventral stream. J. Cogn. Neurosci. 25, 1723–1735. 10.1162/jocn_a_00419 23662861 PMC3767967

[B37] Tyler L. K. Moss H. E. (2001). Towards a distributed account of conceptual knowledge. Trends Cogn. Sci. 5, 244–252. 10.1016/S1364-6613(00)01651-X 11390295

[B38] van der Maaten L. Hinton G. (2008). Visualizing data using t-SNE. J. Mach. Learn. Res. 9, 2579–2605. Available online at: https://www.jmlr.org/papers/volume9/vandermaaten08a/vandermaaten08a.pdf

[B39] Van Rossum G. Drake F. L. (2009). Python 3. Scotts Valley, CA: CreateSpace.

[B40] Viganò S. Piazza M. (2020). Distance and direction codes underlie navigation of a novel semantic space in the human brain. J. Neurosci. 40, 2727–2736. 10.1523/JNEUROSCI.1849-19.2020 32060171 PMC7096136

[B41] Vigliocco G. Vinson D. P. Lewis W. Garrett M. F. (2004). Representing the meanings of object and action words: the featural and unitary semantic space hypothesis. Cogn. Psychol. 48, 422–488. 10.1016/j.cogpsych.2003.09.001 15099798

[B42] Winblad B. Palmer K. Kivipelto M. Jelic V. Fratiglioni L. Wahlund L.-O.. (2004). Mild cognitive impairment—beyond controversies, towards a consensus: report of the International Working Group on Mild Cognitive Impairment. J. Intern. Med. 256, 240–246. 10.1111/j.1365-2796.2004.01380.x 15324367

