# Supplementary material for: Moving in Semantic Space in Prodromal and Very Early Alzheimer's Disease: An Item-Level Characterization of the Semantic Fluency Task
Source: Front Psychol. 2022 Feb 21;13:777656. doi: 10.3389/fpsyg.2022.777656 (PMC8899331; doi:10.3389/fpsyg.2022.777656)
Supplement: Supplementary file 1 [file Data_Sheet_1.PDF]

Supplementary Material

1 SUPPLEMENTARY TABLES AND FIGURES

1.1 Tables

Table S1. Binomial logistic regression models comparing AD-patients with combined aMCI- and control groups.

Model	$\tilde{\chi}^2$	Pseudo R^{2+}	BIC	AIC	*p*
Model 1	34.32	0.52	66.26	58.98	$<.001$
Number of words	26.92				$<.001$
Age	5.37				.021
Model 4	40.94	0.60	64.07	54.35	$<.001$
Number of words	20.79				$<.001$
Age	5.49				.019
Adjusted returns	6.62				.010

Note: $^+$ Nagelkerke's Pseudo R^2 is used.
Abbrevations: BIC= Bayesian information criterion, AIC = Akaike information criterion

Table S2. Model 4. Binomial logistic regression analysis on belonging to a group (healthy controls combined with aMCI compared to AD group) with returns divided by the used subcategories, the number of words, and age as independent variables.

	B	S.E	Wald	Exp(B)	95% C.I	
					L	U
Intercept	-2.54	4.22	0.36	0.08		
Adjusted returns	-0.01	0.04	5.60*	0.91	0.84	0.98
Number of words	-0.33	0.09	12.89***	0.72	0.60	0.86
Age	0.12	0.06	4.74*	1.13	1.01	1.27

Note: Reference category is the AD group. Adjusted returns variable is multiplied by hundred for easier interpretation. Abbreviations: aMCI = amnestic Mild Cognitive Impairment, AD = Alzheimer's dementia, B = unstandardized beta co-efficient, S.E = standard error, Exp(B) = odds ratio, C.I = confidence interval, L=lower bound, U=upper bound. $^*p<.05$, $^{**}p<.01$, $^{***}p<.001$

Table S3. Classification amounts and rates (%) for Models 1 and 4 presented as a confusion matrix.

Model 1

	Predicted		
Observed	Non-AD	AD	Correct (%)
Non-AD	61	5	92.4
AD	7	11	61.1
Overall (%)	81.0	19.0	85.7

Model 4

	Predicted		
Observed	Non-AD	AD	Correct (%)
Non-AD	62	4	93.9
AD	7	11	61.1
Overall (%)	82.1	17.9	86.9

Abbreviations: Non-AD = combined healthy and aMCI groups, AD = Alzheimer's dementia

1.2 Figures

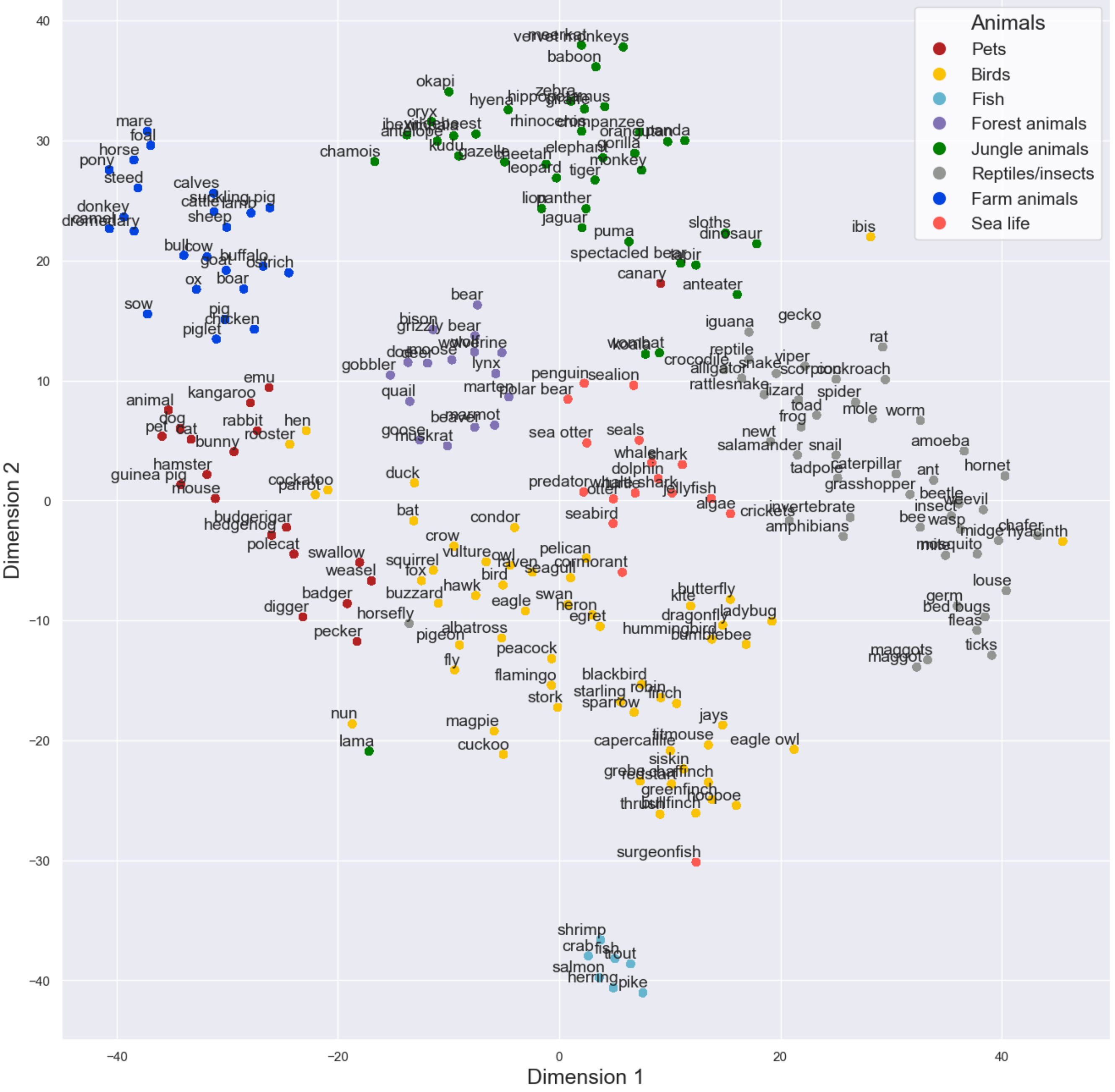

Figure S1. t-SNE mapping with perplexity value 10. Color coding is based on the visually inspected sub-category structure from the final t-SNE model (Figure 1).

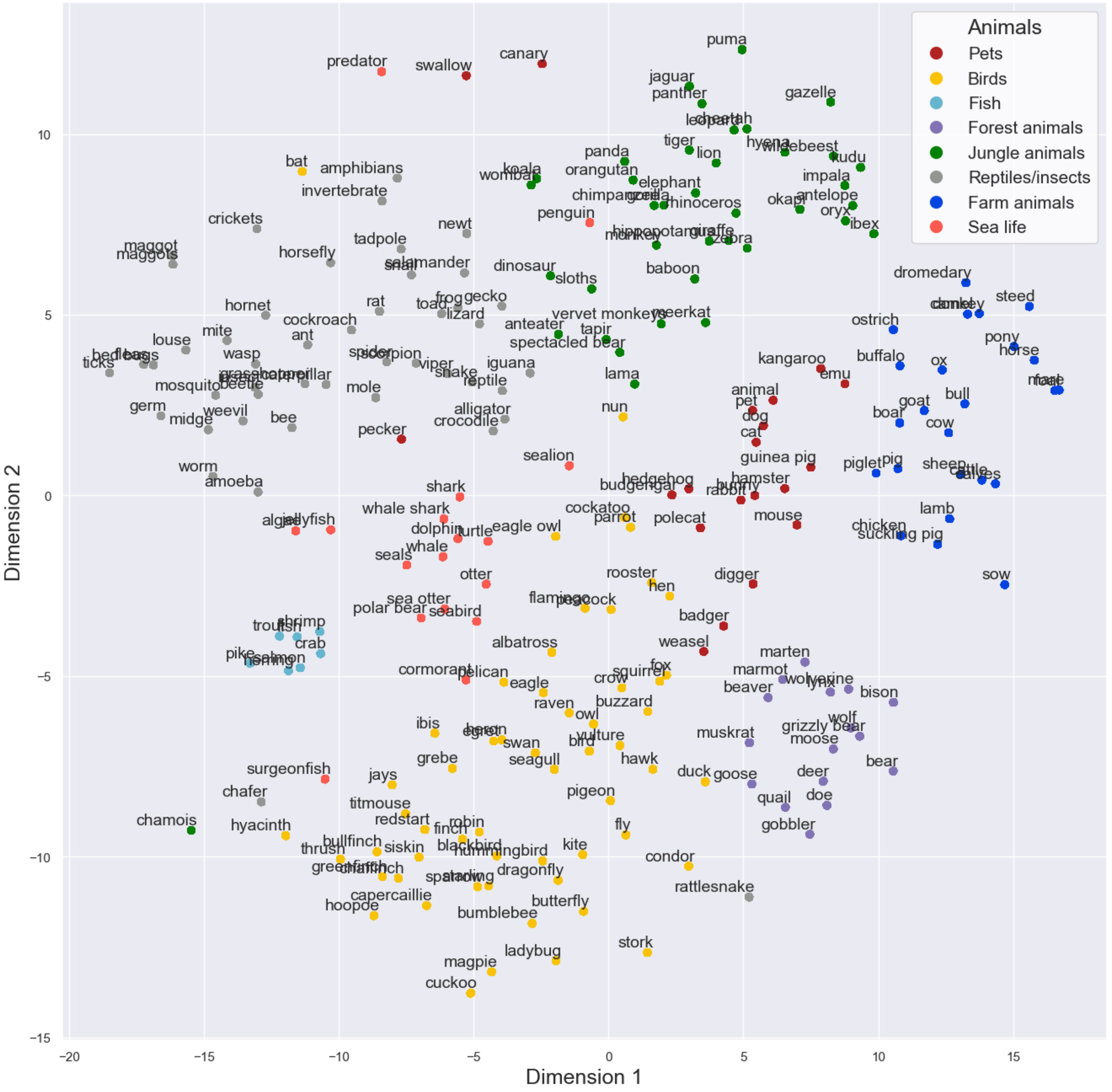

Figure S2. t-SNE mapping with perplexity value 25. Color coding is based on the visually inspected sub-category structure from the final t-SNE model (Figure 1).

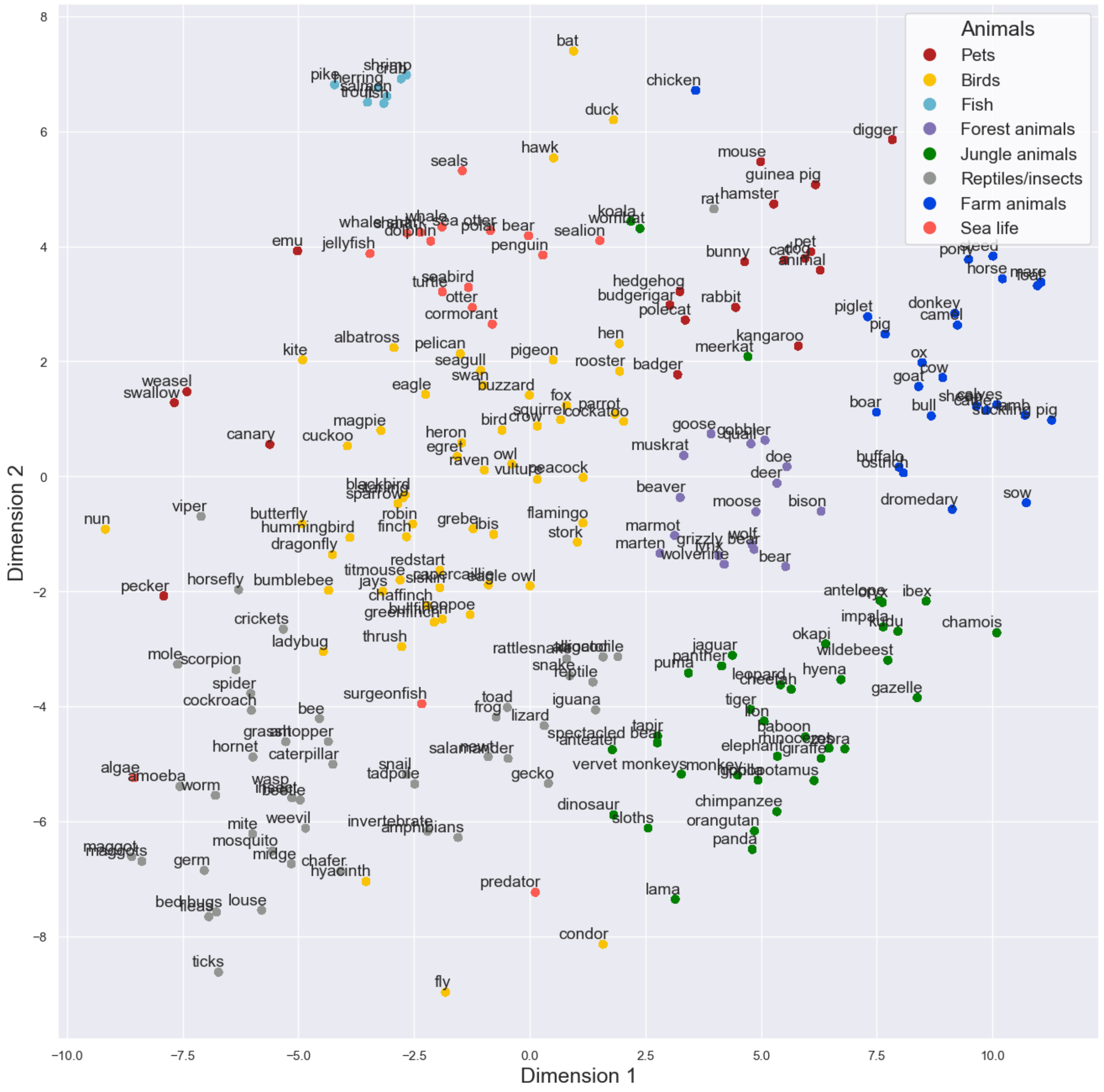

Figure S3. t-SNE mapping with perplexity value 40. Color coding is based on the visually inspected sub-category structure from the final t-SNE model (Figure 1).

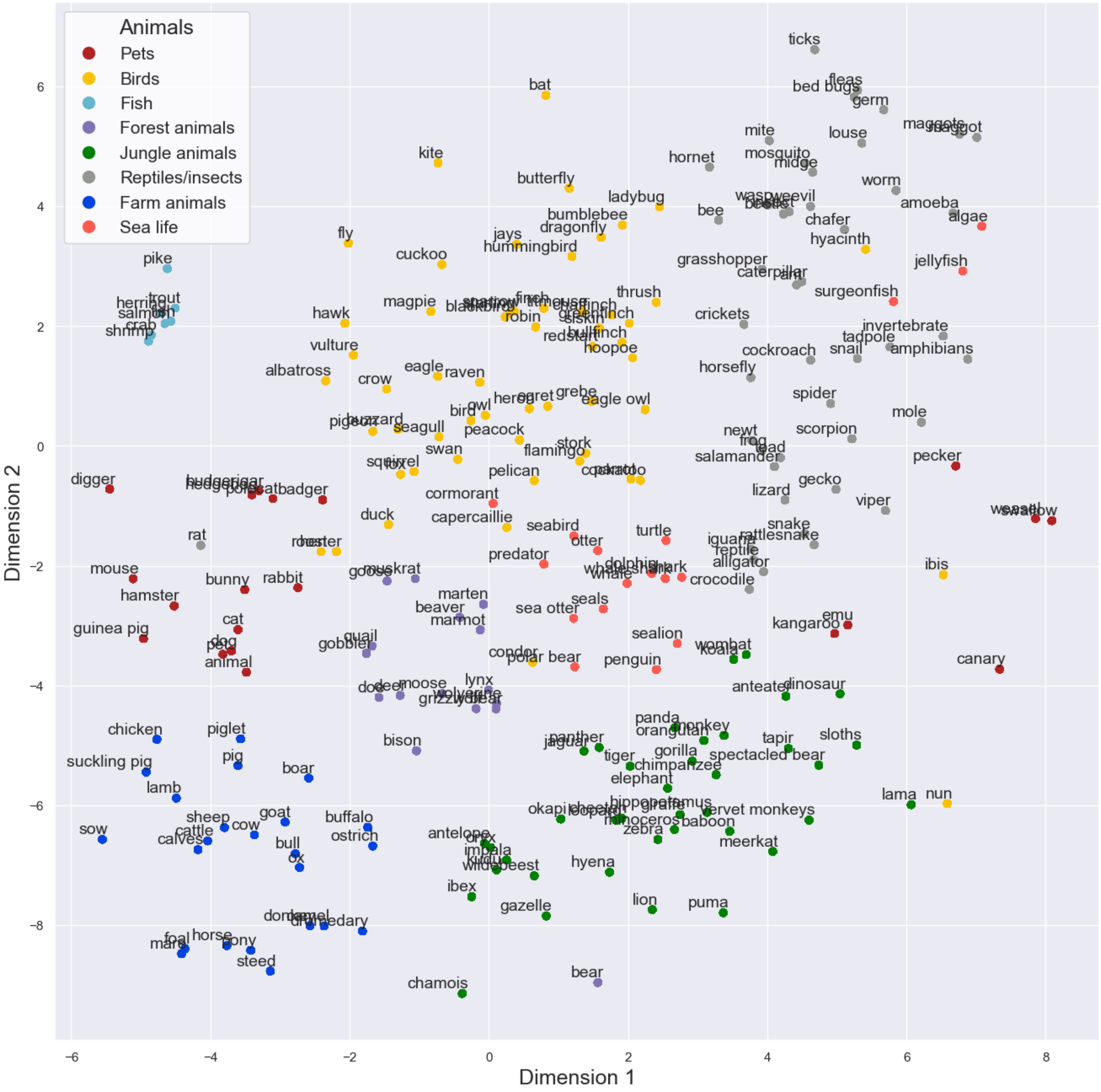

Figure S4. t-SNE mapping with perplexity value 50. Color coding is based on the visually inspected sub-category structure from the final t-SNE model (Figure 1).

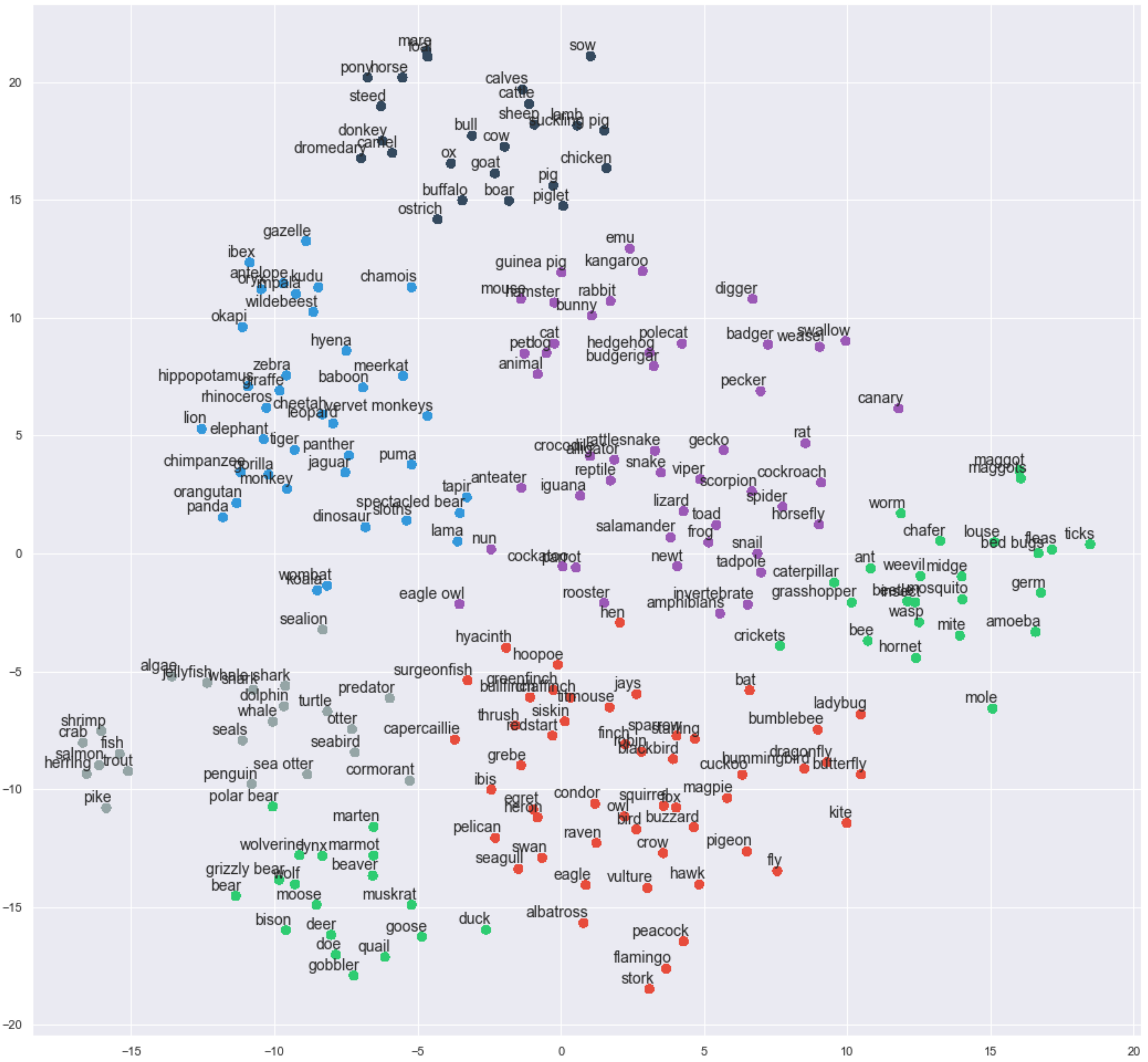

Figure S5. K-means cluster solution based on the final t-SNE model inspected with Python 3.7 (Van Rossum & Drake, 2009) using the package sklearn.cluster (Pedregosa et al., 2011). The optimal number of clusters (k=7) was evaluated using a silhouette score. The estimated interrater reliability measured with the intraclass correlation coefficient between the manually inspected and k-means cluster structures was 0.82 , $p < .001$, with 95 % CI 0.758 - 0.865, which suggests good agreement between our manual labelling and k-means clustering (Koo & Li, 2016).
